# The Past, the Present, and the Future of Hospitals

**Published:** 1886-11-27

**Authors:** Sydney Waterlow

**Affiliations:** Treasurer of St. Bartholomew's Hospital, Bart.


### THE PAST, THE PRESENT, AND THE FUTURE OF HOSPITALS.

By Sir SYDNEY WATERLOW, Bart., Treasurer of St. Bartholomew's Hospital.

(Margin note: The Association and the Sunday Fund. Union is Strength.)

SIR SYDNEY WATERLOW, who received a most cordial welcome from the crowded audience at the Conversazione last week, said, he had once, in his early days, hoped his father would have educated him for the medical profession, but the *res angusta domi* did not permit. Sir Andrew Clark had spoken of the advantages of the Hospitals Association. He endorsed every word he had said. The hospitals of the metropolis had been very much helped, and their management greatly improved, by the attempts which had been made during the past ten years to bring about friendly communion among them. Those attempts had been greatly strengthened by the founding of the Hospitals Association. The establishment of Hospital Sunday, with all its organisations, led to the conviction that a greater co-operation and interchange of views among hospitals was desirable. The Hospital Sunday Fund brought together on its Council not only those who pleaded for its funds, but a large number of gentlemen engaged in the management of large medical charities. More than a hundred medical charities now claimed and received assistance from the fund. These institutions had been classified for the purposes of the distribution of the fund, and this very fact facilitated a comparison of their economy and management, and so brought about an interchange of views.

(Margin note: Nursing, Old and New.)

Sir Sydney then adverted to the superior character of the nursing now prevailing at the hospitals. The growth of intercommunication among them had been proceeding *pari passu* with this improved nursing. The sick poor had derived great advantage from the higher intelligence and character of those who were now conducting the nursing of the hospitals. He did not speak from his own experience only, but also from that of the ablest physicians and surgeons of the time. Many, perhaps, might be surprised at the very rapid improvement which had been effected even in so brief a period as the past ten years. He instanced some of those improvements which had come under his own knowledge during his chairmanship of the Central London Sick Asylum and his treasurership of St. Bartholomew's Hospital.

(Margin note: Ten Years Ago.)

In 1877 they had no ward-maids at Bartholomew's, and, generally speaking, there were none at the other hospitals. All the work had to be done by the nurses. It could scarcely be expected, when nurses were called upon to clean pots and scrub floors, that ladies accustomed to the refined elegancies of life could be induced, as they now were, to enter upon the arduous duties of sick nursing. After pointing out the benefits which had accrued to the sick poor from the appointment of a relatively larger number of skilled nurses, Sir Sydney expressed his belief that the public, on the whole, were satisfied that their money was well and judiciously expended. He never saw so many examples of unselfish devotion as there were to be found among hospital sisters and nurses. There were in London hundreds of ladies, coming from homes of comparative luxury, who thought that life without an object was not worth living, and so they had come forward to nurse the sick. All honour to them for their noble devotion. Those who wished to see examples of this personal sacrifice might go into any of the large hospitals, and there they would find as many as they might like to see.

(Margin note: The Pioneers and Heroines of the New System.)

After referring to the noble work of Florence Nightingale and to Mrs. Wardroper, to whom St. Bartholomew's was greatly indebted, Sir Sydney expressed the hope that, through the Hospitals Association and the influence which it would yet exercise over the public mind, the time was not far distant when people would as little think of engaging a nurse who could not produce a certificate of competency as they would now think of calling in an unqualified medical man. Good nursing was not second to, but ran concurrently with, good medical and surgical advice. The best medical advice was almost useless unless there was an intelligent nurse to carry out the physician's or surgeon's instructions.

(Margin note: Medical Schools and Patients.)

Referring to medical schools, he said he considered the school and the hospital were more or less in the position of man and wife, and each as indispensable as the other. He was quite willing to admit, and he had often contended before, that if there were any question on which there was the least clashing of interests, the patients should undoubtedly come first. But the school should follow so immediately second, that one could scarcely distinguish that it was in the rearguard. The system of teaching in the hospital medical schools was of great advantage to the patients, and of even more advantage to the community generally. When the Government established the infirmaries in connection with the Poor-law system, they put a clause in the statutes prohibiting young men from attending these infirmaries as students. It seemed to him that, seeing these institutions were supported by the rates, the public had the right, and should insist upon their right, to demand that they should be used as schools for the training of medical juniors in every practicable way. He looked forward to the time when all this would be changed.

Convalescent and Ambulance Agencies.

Another great improvement of the past fourteen or fifteen years was the rapid increase in the number of convalescent homes in connection with the hospitals. Ten years ago these homes were comparatively few; there were not more than five or six which were in a position to ask for assistance from the Hospitals Sunday Fund. Now that number had been increased fourfold. Sir Sydney next dwelt upon the vast benefit of these institutions to poor men and women who were just leaving the hospitals. A few weeks at a convalescent home enabled them to regain sufficient strength to battle successfully with the unsanitary condition of their homes, and to pursue their daily work with adequate energy. Sir Sydney then related his experience of the ambulance system as he had seen it in New York. Many and many a time had limbs and even life been lost from the manner in which injured persons were bent up in "growlers" and trundled over the stones, or otherwise hustled mercilessly to the hospital.

The value of Co-operation.

Sir Sydney then urged the value of co-operation and constant discussion in all questions of hospital administration. He had referred to the happiness which many persons felt in taking part in hospital work; that privilege he deeply valued himself. With the support of the public he believed their work would be easy. No one present would say that the public were not, on the whole, satisfied with the management of the hospitals. The great fault was that the public did not know enough about them. The more they knew, the greater would be their readiness to increase their already liberal help. He believed that subscriptions would be largely increased with the increase of knowledge of the subject, and that could be best accomplished by each one making himself familiar with the history and work of the hospitals.

### Recent Novelties in Sick-Room Appliances.

Hospital Novelties.

